# Cytotoxicity and Antibacterial Activity of Protonated Diallylammonium Polymers: Influence of End Groups and Molecular Weight

**DOI:** 10.3390/ijms26041501

**Published:** 2025-02-11

**Authors:** Larisa M. Timofeeva, Yulia A. Simonova, Ivan V. Eremenko, Marina P. Filatova, Maxim A. Topchiy, Nataliya V. Kozobkova, Margarita O. Shleeva, Mikhail Yu. Eropkin

**Affiliations:** 1 A.V. Topchiev Institute of Petrochemical Synthesis of Russian Academy of Sciences, 119071 Moscow, Russia; simonova@ips.ac.ru (Y.A.S.); iveremenko@ips.ac.ru (I.V.E.); filatova@ips.ac.ru (M.P.F.); maxtopchiy@ips.ac.ru (M.A.T.); 2 Federal Research Centre “Fundamentals of Biotechnology” of Russian Academy of Sciences, 119071 Moscow, Russia; natalia.cosolapova@gmail.com (N.V.K.); margoshleeva@gmail.com (M.O.S.); 3 Ministry of Heath of Russian Federation Smorodintsev Research Institute of Influenza, 197376 Saint-Petersburg, Russia

**Keywords:** free radical polymerization, protonated polydiallylamines, end groups, antibacterial activity, cytotoxicity, eukaryotic cells

## Abstract

A series of antimicrobial protonated diallylammonium polymers, poly(diallylammonium trifluoroacetate) (PDAATFA), were synthesized by classical polymerization, using an especially elaborated method for preparation of polymers with low molecular weight (MW), and by RAFT polymerization, with different end groups in a range of MW values of (8–43) × 10^3^ g∙mol^−1^. Cytotoxicity relative to eukaryotic cells (epithelioid lines A-549 and MA-104) and bactericidal activity of the polymers (relative to *Pseudomonas aeruginosa* and *Staphylococcus aureus*) are investigated. The effect of the end groups and MW on toxicity and bactericidal activity is shown. Dependence of the activity and, most of all, cytotoxicity on MW is preserved even at a small difference in MW values in the MW range of (18–40) × 10^3^ g·mol^−1^. A clear dependence of the studied properties on the nature of the terminal group is revealed. Sulfate -O-S(=O)_2_-O¯ end group has a noticeable effect on the bactericidal efficiency and smaller influence on toxicity, while dithiocarbonyl end group -S-C(=S)-O-CH_2_-CH_3_ has a significant effect on efficiency and especially toxicity, drastically increasing the latter. Overall, based on the results obtained, polymers PDAATFA of low MW are considered promising antimicrobial agents for the creation of new transdermal drugs.

## 1. Introduction

Antimicrobial synthetic polymers, including synthetic mimics of naturally appearing host-defense antimicrobial peptides, have attracted increasing attention from researchers over the last two decades, mainly in relation to the threat of bacterial resistance to antibiotics that developed in recent years. Biocompatible antimicrobial polymers are considered hopeful candidates for antimicrobial therapeutics since the probability for the resistance development seems to be minimal because of their nonspecific action, unlike antibiotics, which act on the selected targets [1,2,3,4,5,6,7,8,9,10]. Different ways have been proposed to obtain biocidal but low-toxicity polymers [1,2,3]. One of them is the synthesis of degradable macromolecules, which are toxic as a whole molecule, while the units of the polymeric backbone itself are not biocidal [2,10]. One of the used concepts was combining hydrolytically degradable antimicrobial polymers (in particular, polymers based on polyionenes) with the satellite end groups, which deactivate polymers in the surrounding environment without causing environmental problems [10]. Several approaches have been suggested for the synthesis of mimics of host-defense antimicrobial peptides [4,5,6,7,8,9]. They include ROMP- and RAFT-based guanidinium-containing polymers [6], polymers based on asymmetrically disubstituted itaconates [7], and polymers based on polymethacrylates and poly(vinyl ether)s [8,9]. In these investigations, it has been shown that the achievement of balance between the hydrophobic properties of the (co)polymer and its functional hydrophilic characteristics is a key factor for the combination of antimicrobial activity and low toxicity [4,5,6,7,8,9].

Among antimicrobial polymers, water-soluble protonated secondary/tertiary diallylammonium polymers (PDAAs) based on protonated diallylammonium salts are of interest [11,12]. The behavior and many properties of PDAAs differ from those of their quaternary analogs and conventional cationic quaternary polyamines due to the protonated ammonium group in the links. Moreover, the combination of hydrophobic pyrrolidinium structure with hydrophilic ammonium group might endow polymers with new properties that differ from those of the quaternary counterparts. This was confirmed by PDAAs featuring high nonspecific antimicrobial efficacy relative to some hospital pathogens [13], including rare activity against mycobacterium *M. tuberculosis* [14], unlike quaternary polymers of this series [15]. It has been shown using the example of *M. smegmatis* cells that protonated ammonium groups of PDAA are mainly responsible for a violation of the structure and integrity of the outer membrane of the cell and, as a result, cell death [16]. Importantly for practical applications, these polymers exhibit quite high biocidal activity in serum and 0.01M/0.1M salt solution, i.e., until the macrochain retains some positive charge and there are some active nitrogen centers unscreened by counteranions. The secondary PDAA retains quite high biocidal efficiency in an aqueous alkaline solution, in particular, in the presence of an equimolar amount of NaOH, where this polymer salt exists as a water-compatible polybase [13].

The monomeric unit of protonated PDAA is the 3,4-methylene-substituted pyrrolidinium ring. With that in mind, we may consider PDAA polymer as protonated poly((3,4-methylene)pyrrolidine). It is known that pyrrolidine (Pyr) and its C-substituted derivatives are used as scaffold for bioactive compounds (antitumor, antidiabetic, antibacterial, etc.) [17,18]. Therefore, a question about the cytotoxicity of PDAAs and selectivity of their action is, as for other polymer biocides, a question about their polymeric nature’s influence on both cytotoxicity and antimicrobial activity. We have shown via an in vitro study on mammalian cells that two groups of PDAA polymers can be distinguished as promising for application [19]. The first is a group of PDAAs with a sufficiently large MW (more than 50 × 10^3^ g·mol^−1^) which have strong biocidal activity [13]; these PDAAs’ biocidal concentrations (in particular, relative to *Staphylococcus aureus*) are an order of magnitude lower than their concentrations, thus causing strong toxic effects on mammalian cells [19]. These PDAAs seem to be promising as disinfectants. The samples of low MW (18 × 10^3^ g·mol^−1^ and lower) are also of interest. Their biocidal activity is slightly higher (selectivity about 1.16 relative to *Staphylococcus aureus*) than their moderate cytotoxicity [19]. In this paper, we focus on the study of the bioactive properties of the PDAAs of low and medium MW, namely on the possible effect of different end groups on both toxicity and bactericidal efficiency of the polymers.

With the development of the controlled radical polymerization method with a reversible addition–fragmentation chain transfer (RAFT) mechanism, it became possible to synthesize polymers with variable functional properties due to the introduction of end groups of the RAFT agent [20,21,22,23]. It allows us to evaluate the effect of the end groups in RAFT polymers [23], in particular, in regard to their antimicrobial activity/toxicity [24]. However, our study of applicability of the RAFT method for radical polymerization of DAATFA with a significant kinetic contribution of the efficient chain transfer to the monomer (that noticeably affects molecular weight and polydispersity of the polymers) has shown that the choice of water-soluble RAFT agents to control polymerization is extremely limited in this case [25]. Therefore, in the present work, we used a procedure of free radical polymerization elaborated especially for synthesis PDAA polymers with low MW that allows us to obtain samples with variable end groups [26].

## 2. Results and Discussion

### 2.1. Synthesis and Characteristics of Polymers

**Scheme 1:**
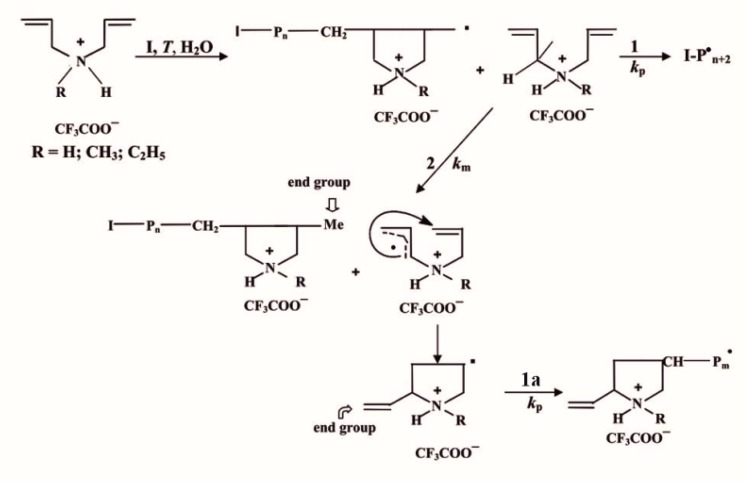
Cyclopolymerization of protonated diallylammonium monomers: paths 1 and 1a —chain propagation, *k_p_*; and path 2—chain transfer to monomer, *k_m_*, with subsequent transformation of the diallyl transfer radical into a chain propagation radical via the intramolecular cyclization following by chain propagation, *k_p_*.

PDAAs were first synthesized by free radical cyclopolymerization of protonated salts—trifluoroacetates of diallylammonium monomers (Scheme 1) [11,12]. This polymerization occurs with efficient chain transfer to monomer the due to the transformation of the protonated diallyl transfer radical into a chain propagation radical via the intramolecular cyclization, with the formation of the end vinyl group being the distinct feature of the process. The degree of polymerization depends significantly not only on the concentration of the initiator, but also on the temperature, which influences the probability of the chain transfer reaction. (More exactly, the constant of the chain transfer to monomer is determined by the Δ*E_a_*/*RT* factor, where Δ*E_a_* is the difference in activation energies of the chain propagation and chain-transfer-to-monomer reactions) [11,12,26].

To prepare polymer samples of PDAA with a low degree of polymerization and different end groups, the method has been elaborated for radical polymerization in excess of initiators, and the influence of temperature and initiator concentration has been taken into consideration [27].

It has been shown (using NMR, IR spectroscopy, and elemental analysis) that in the case of excess of initiator, the characteristic reaction (**2**) of the effective chain transfer to the monomer (and chain propagation reaction as well; see Scheme 1) is largely kinetically suppressed by the reaction (**3**) of macroradicals with the primary radicals of the initiator (as seen below):

(**2**) I–P_n_^∙^ + M → I–P_n_–CH_3_ + CH_2_=CH–P^∙^—chain transfer to monomer;

(**3**) I–P_n_^∙^ + I^∙^ → I–P_n_–I—interaction of I^∙^ with a macroradical.

Accordingly, with the excess of initiator and a decrease in the molecular weight of polymers, the relative number of characteristic terminal vinyl groups diminishes, and the end groups formed by the termination of macroradicals by the primary radicals of the initiator become predominant [27].

In the present paper, radical polymerization of diallyl monomer salt, diallylammonium trifluoroacetate (DAATFA), was carried out in excess of initiators, ammonium persulfate (APS) and 4,4′-azobis(4-cyanovaleric acid) (ACVA), so as to obtain poly(diallylammonium trifluoroacetate) (PDAATFA). As follows from Table 1, by varying the initiator concentration and temperature, a series of PDAATFA polymer samples were obtained in the MW range of (8−43) × 10^3^ g·mol^−1^. The results in Table 1 evidence the significant effect of temperature growth on the increase in probability of the chain transfer to the monomer. An increase in the initiator concentration and the growth in contribution of the chain transfer to the monomer with increasing temperature both lead to a similar decrease in MW (compare samples P3 and P4).

**Figure 1:**
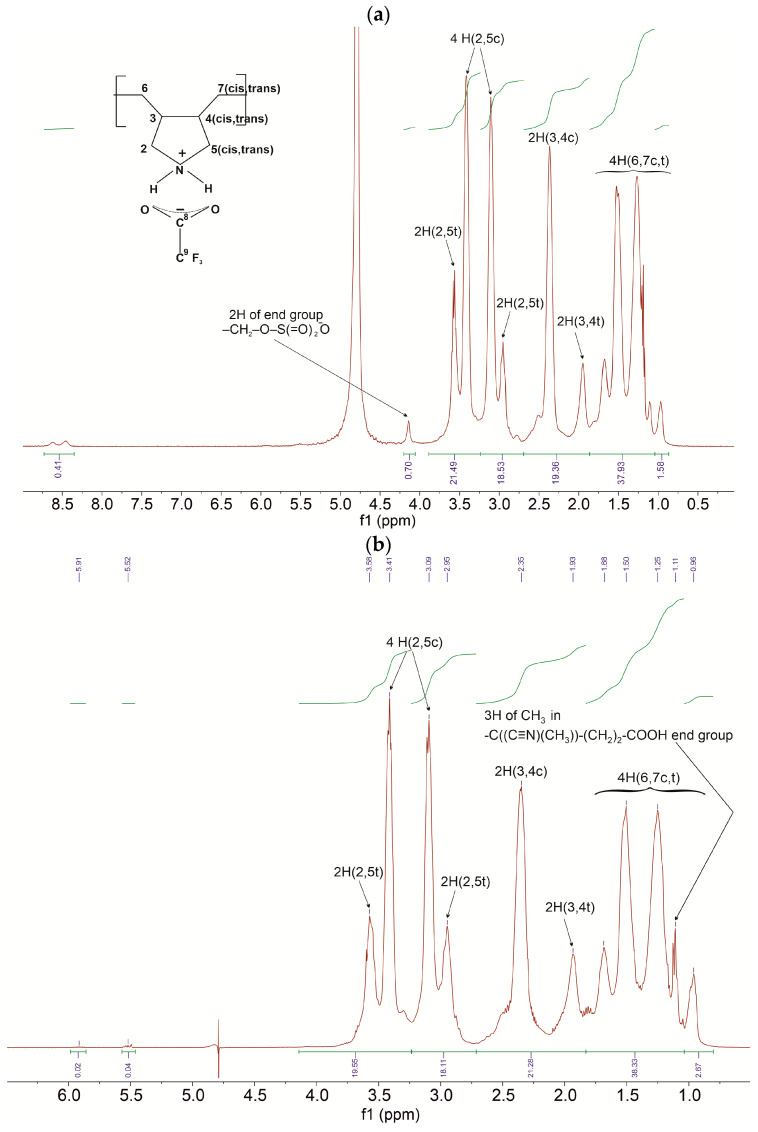
^1^H NMR spectra of polymers PDAATFA, Bruker AVANCE III HD (400 MHz ^1^H), D_2_O. Signals due to hydrogen atoms of macrochain are assigned using the two-dimensional HSQC spectrum [28]: (**a**) sample P4 and (**b**) sample P5.

**Figure 2:**
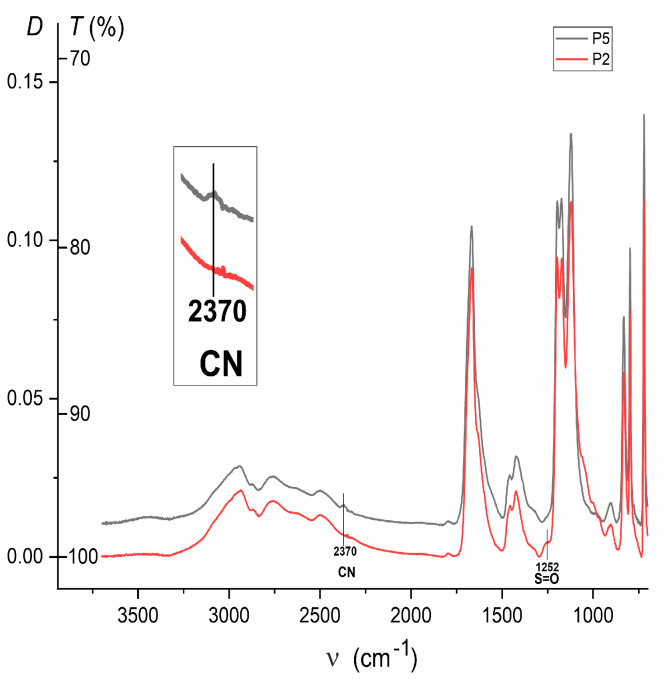
Comparison of IR-Fourier spectra of P2 and P5 samples. Spectra are presented in the “*D*–ν” coordinates, where *D* is dimensionless optical density (linear scale), *T* is transmission coefficient scale (nonlinear scale), and *D* = lg(100/*T*) (see Section 3).

In the case of polymerization with an excess of initiator, small or poorly detected signals of end vinyl groups and the formation of terminal groups of primary radicals of the initiator are confirmed by the NMR ^1^H and IR-Fourier spectra of the samples prepared with excess of initiators (10^−1^ mol·L^−1^) (Figure 1 and Figure 2; Table 1).

In the spectrum of sample P4 prepared with APS (Figure 1a), the signal in the region of 4.15 ppm should be assigned to H atoms of -CH_2_-O-S(=O)_2_-O¯ end sulphate group, while signals due to the end vinyl groups (region 5.5–6.0 ppm) are poorly detected. In the spectrum of sample P5 prepared with ACVA (Figure 1b), weak signals of the end vinyl group are registered in the region 5.5–6.0 ppm, while signals at 1.11 ppm (triplet), superposed at the signals of H (6,7 c,t), should be assigned to H atoms of CH_3_ group in the -C((C≡N)(CH_3_))-(CH_2_)_2_-COOH end group.

The latter is confirmed by the IR-Fourier spectrum of the sample P5 in comparison with the spectrum of the sample P2 (Figure 2). The spectrum of P5 clearly shows a weak band 2370 cm^−1^ from the valence vibrations of the C≡N bond of the -C((C≡N)(CH_3_))-(CH_2_)_2_-COOH end group, which is absent in the spectrum of P2. Meanwhile, in the spectrum of P2, there is a weak band 1252 cm^−1^ from the valence vibrations of the S=O bond of -CH_2_-O-S(=O)_2_-O¯ end group, which is absent in the spectrum of P5. The bands of valence vibrations of nitrile groups lying in the region of 2350–2420 cm^−1^ have a low intensity, 2.5 times lower than the bands of valence vibrations of bonds S=O 1252 cm^−1^.

**Table 1:** Molecular characteristics of PDAATFA samples obtained with an increased concentration of the initiators: intrinsic viscosity [*η*]; molecular weight, *M_D_*; and *M_η_* for PDAATFA samples in 1 M NaCl at 298 K.

Polymer Sample	Polymer	I	[I], mol·L^−1^	*T*, °C	[*η*], cm·g^−1^ (k′)	*M_Dη_* × 10^−3^, g·mol^−1^ (Based on A_0_)	*M_η_* × 10^−3^, g·mol^−1^ (from M-K-H)
P0	PDAATFA	APS	2 × 10^−2^	50	13.0 ± 0.8	43 ± 1	42.8 ± 4.9
P1	PDAATFA	APS	4 × 10^−2^	40	12.8 ± 0.8	40 ± 2	41.6 ± 4.8
P2	″	″	10^−1^	50	8.1 ± 0.4 (k′ = 0.439)	17.9 ± 0.9	17.8 ± 1.6
P3	″	″	4 × 10^−2^	50	10.0 ± 0.6 (k′ = 0.398)	27 ± 2	26.3 ± 2.9
P4	″	″	10^−1^	40	10.0 ± 0.8 (k′ = 0.429)	28 ± 1	26.3 ± 3.9
P5	″	ACVA	10^−1^	70	9.3 ± 0.4 (k’ = 0.40)	**-**	23.0 ± 1.8
P6	″	″	4 × 10^−2^	70	11.8 ± 0.5 (k′ = 0.496)	**-**	35.8 ± 2.8
P8 ^a^	″	ACVA	5 × 10^−3^	70	5.1 ± 0.5	8.0 ± 0.5	7.6 ± 1.4

^a^ Sample P8 was prepared in this work by RAFT polymerization in the presence of RAFT agent xanthate at a concentration of 1.5 × 10^−2^ mol·L^−1^ (Section 3).

### 2.2. Toxicity of Tested Polymers

Investigations of cytotoxicity were performed using in vitro method on cell cultures. Investigations of cytotoxicity by in vitro method are an alternative to classical tests on experimental animals. All data obtained over 30 years indicated that the parameters of basal toxicity in vitro are very similar irrespective of the species and tissue origin of the established mammalian and human cell lines and are universal for all types of cells [29,30,31]. In this work, permanent (established) cell lines A-549 (epithelioid line of human lung carcinoma) and MA-104 (epithelioid line of green monkey kidney) were used for the study. The dose of the substance in the well was determined at which 50% destruction of the cellular monolayer, CTD_50_, was observed (methodology of toxicity research is presented in Section 3). The chosen polymer concentrations were based on the results of previous studies in which samples were investigated in a wide range of concentrations [19].

**Figure 3:**
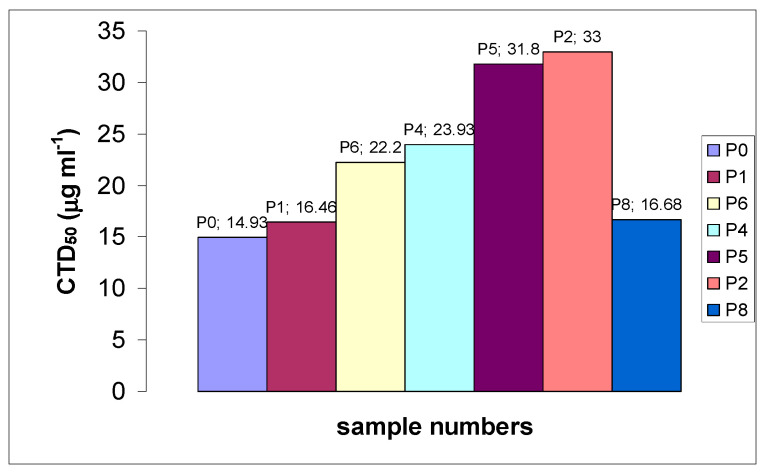
Toxic concentration, CTD_50_ (relative to A-549 cell line), which was observed for the polymer samples (characteristics of samples are provided in Table 2).

The results of toxic-effects investigations of the secondary polydiallylamines PDAATFA with variable MWs and different end groups are presented in Table 2 and Figure 3. Analysis of the obtained toxicity data shows that the dependence of toxicity on MW (increase along with MW) is observed for polymers of medium and low MW. As shown for PDAAS in the MW range (40–118) × 10^3^ g∙mol^−1^, their CTD_50_ values (14–19 μg·mL^−1^ for A-549 cell line, and 25–31 μg·mL^−1^ for MA-104 cell line) vary little in this MW range [19]. However, pyrrolidine and the monomeric salt pyrrolidinium trifluoroacetate (PyrTFA), which models the monomer unit of PDAA polymers, exhibit a weak cytotoxic effect, and their CTD50 is more than two orders of magnitude higher (1570 ± 346 and 3840 ± 1070 μg·mL^−1^, accordingly, for A-549 cell line) than that of all polymers studied [19]. Thus, it may be concluded that the high toxic effect of polymers is due to the cooperative action of the hydrophobic content and the total charge of the polycation.

One may partially shed light on the mechanism of toxic action of the polymers if one takes into consideration the method of toxicity testing [32]. According to widely accepted opinion, tetrazolium (MTT) is mainly restored by mitochondrial and partly cytoplasmic dehydrogenases; therefore, it serves as an integral indicator of the intensity of cellular respiration. It is known that the suppression of the cellular respiration, i.e., the transmembrane potential (TM) of a cell, is due to the effect of polymers on ion flows through the cell membrane. With the growing MW and total charge of a polycation, its influence on cell ion flow increases. The counterions affect cell-ion flows as well. A decrease in TM potential value over extended treatment time should lead to cell death because of the inhibition of general bioenergetic processes. When studying the antimicrobial action of protonated PDAATFA polymers on M. smegmatis, we have shown that the influence of both the protonated PDAATFA and quaternary poly(diallyldimethylammonium chloride) on TM of *M. smegmatis* causes the suppression of the TM potential [16].

**Table 2:** Cytotoxic concentration (CTD_50_) ^a^ of aqueous solutions of polymer samples, treatment time 24 h, C_cell_ = 10^5^ CFU (of tested eukaryotic cells).

Sample	MW × 10^−3^, g·mol^−1^	End Group	CTD_50_, μg·mL^−1^ Relative to A-549	CTD_50_, μg·mL^−1^ Relative to MA-104
P0	43.0	CH_2_=CH-; CH_3_	14.93 ± 0.36	25.44 ± 1.14
P1	41.6	CH_2_=CH-; CH_3_	16.46 ± 0.92	24.87 ± 0.10
P2	17.8	-O-S(=O)_2_-O¯	33.0 ± 2.97	43.65 ± 1.17
P4	26.3	-O-S(=O)_2_-O¯	23.93 ± 1.5	31.14 ± 1.81
P5	23.0	-C((C≡N)(CH_3_))-(CH_2_)_2_-COOH	31.68 ± 1.75	-
P6	35.8	-C((C≡N)(CH_3_))-(CH_2_)_2_-COOH	22.0 ± 3.41	-
P8	8.0	-S-C(=S)-O-CH_2_-CH_3_; -CH_2_-COOH	16.68 ± 0.42	25.80 ± 1.08

^a^ See Section 3.

Comparison of samples with close MW and different terminal groups (P4 and P5), or close toxicity, but different MW and terminal groups (P4 and P6) allows us to conclude that sulphate terminal groups -O-S(=O)_2_-O¯ contribute to greater toxicity of the polymer compared to terminal groups -C((C≡N)(CH_3_))-(CH_2_)_2_-COOH.

**Figure 4:**
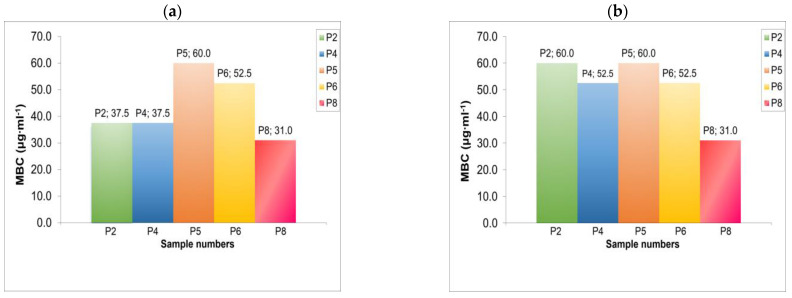
MBC values for tested polymers relative to (**a**) *Staphylococcus aureus* and (**b**) *Pseudomonas aeruginosa*; treatment time 24 h; C_cell_ = 10^5^ CFU. MW values of samples are provided in Table 3.

The high cytotoxicity of RAFT-PDAATFA (sample P8 with dithiocarbonyl -S-C(=S)-O-CH_2_-CH_3_ end group) with the lowest molecular weight of the tested polymers was unexpected. It has shown toxicity comparable to that of samples P0 and P1 with a MW five times higher. The effect of the dithiocarbonyl group on PDAATFA toxicity turned out to be more significant than on antimicrobial activity (see Table 3 and Figure 4). Thus, in the case of a polymer with a small MW, the polar lipophilic group has a strong cytotoxic effect on eukaryotic cells. This result does not coincide with the data on the weak influence of -S-C(=S)-Z (Z = S(CH_2_)_11_CH_3_, SCH_2_CH_3_) groups on the hemolytic activity (as a measure of toxicity) of the polymethacrylates [24]. However, it would not be correct to compare the results obtained for such different cells as eukaryotic cells and erythrocytes, as well as for different exposures.

Higher toxicity to human malignant cells than to non-transformed kidney cells was expected (Table 2). Lower cellular elasticity (low Young’s modules) is a distinguishing feature of cancer cells compared with normal cells [33]. It may lead to the easier compressibility of these cells under the electrostatic action of polycations causing known lateral segregation of molecules of the outer leaflet of cell wall. Nevertheless, importantly, the relations between data on toxicity of polymers in one row are the same for two cell lines (Table 2), thus confirming the conclusions [29,30,31]. However, in comparison with the bactericidal activity data, toxicity values obtained for the MA-104 cell line seem to be more adequate.

### 2.3. Bactericidal Activity of Tested Polymers

The dependence of the antibacterial activity of polymers on their MW was discovered by Ikeda [34], and the effect of MW on the antibacterial activity of various polymers and synthetic mimics of antibiotic peptides was discussed for different polymers [2,3,35,36,37]. From the data presented in Table 3, it is evident that PDAATFA with low MWs still retain a sufficiently high biocidal activity. The results on the activity of samples with a low MW show a dependence on the nature of the terminal group (Table 3 and Figure 4). This is revealed when comparing the MBC of samples P2 and P4 with P5 and P6 against *Staphylococcus aureus*. Polymers P2 and P4 with the end sulphate group -O-S(=O)_2_-O¯ have also shown higher toxicity compared to polymers P5 and P6 with the -C((C≡N)(CH_3_))-(CH_2_)_2_-COOH end group (Table 2 and Figure 3). The terminal dithiocarbonyl group has shown the strongest influence on the antimicrobial activity, significantly enhancing the bactericidal efficiency of the P8 sample.

**Table 3:** Minimal bactericidal concentration (MBC) of polymers relative to *Staphylococcus aureus* and *Pseudomonas aeruginosa*, treatment time 24 h, C_cell_ = 10^5^ CFU ^a^.

Sample	MW × 10^−3^, g·mol^−1^	MBC *Staphylococcus aureus*, μg·mL^−1^	MBC *Pseudomonas aeruginosa*, μg·mL^−1^
P2	17.8	37.5 ± 7.5	60 ± 2
P4	26.3	37.5 ± 7.5	52.5 ± 7.5
P5	23.0	60 ± 2	60 ± 2
P6	35.8	52.5 ± 7.5	52.5 ± 7.5
P8	8.0	31 ± 3.1	31 ± 3.1

^a^ 500 μg·mL^−1^ was the maximal initial concentration of the polymer solutions to be investigated.

PDAATFA polymers have exhibited greater efficiency relative to Gram-positive *Staphylococcus aureus* than to Gram-negative *Pseudomonas aeruginosa*. The same behavior of PDAATFA and tertiary poly(diallylmethylammonium trifluoroacetate) (PDAMATFA) polymers was observed earlier upon investigating the high-MW polymers. For PDAATFA (*M*_w_ 62 × 10^3^ g·mol^−1^), MBC = 1.5 ± 0.3 μg·mL^−1^ relative to *Staphylococcus aureus*, and for PDAMATFA (*M*_w_ 55 × 10^3^ g·mol^−1^), MBC = 7.0 ± 1.0 μg·mL^−1^, while relative to *Pseudomonas aeruginosa*, MBC = 125 ± 7.5 μg·mL^−1^ and MBC = 31 ± 3.1 μg·mL^−1^, accordingly [13]. McDonnell and Russell noted that, as a whole, Gram-negative microorganisms exhibit stronger resistance to antiseptics and disinfectants than Gram-positive ones (with the exception of Gram-positive mycobacteria) [38]. Franklin and Snow related this to the structure of the cell walls (CWs) of these bacteria [39]. As has been evidenced from the data [13] and this investigation, the structure of the ammonium group in polymers with the similar lipophilic characteristics has various influence on the activity relative to bacteria with different structures of CWs.

## 3. Materials and Methods

### 3.1. Materials

Trifluoroacetic acid (TFA, “for synthesis”, ≥99.0%; Merck, Darmstadt, Germany), and radical initiators ammonium persulfate (APS, 99+%, for molecular biology, DNAse, RNAse and protease free, Acros, Geel, Belgium) and 4,4′-azobis(4-cyanovaleric acid) (ACVA, 98.0%; Aldrich, St. Louis, MO, USA) were used without additional purification. Diallylamine (DAA) reagents (for synthesis, 97%; Acros; Geel, Belgium), and solvents hexane and diethyl ether (“analytically pure”, Khimmed; Moscow, Russia) were distilled before use. Chromatographically pure DAA: *T*_b_ = 111–112 °C. ^1^H NMR (Me_2_CO-d_6_): 3.20 (d, 4H, 2α-CH_2_, *J* = 5.89 Hz), 5.12 (m, 4H, 2γ-CH_2_), 5.87 (m, 2H, 2β-CH).

### 3.2. Synthesis

The procedures for obtaining trifluoroacetic salts from monomer DAA were described previously [11,12]. The structures were confirmed by ^1^H NMR spectra (characteristic spectrum is given in [9]). ^1^H NMR for DAATFA (Me_2_CO-d_6_): 3.71 (d, 4H, 2α-CH_2_, *J* = 6.43 Hz), 5.47 (m, 4H, 2γ-CH_2_), 6.00 (m, 2H, 2β-CH).

### 3.3. DAATFA Polymerization

Polymerization of the DAATFA was carried out according to the elaborated method [12,13]. Aqueous solutions of DAATFA, [M] = 2 mol/L, at several concentrations of the APS initiator, [I] = 2 × 10^−2^, 4 × 10^−2^ and 10^−1^ mol/L, and T = 40 and 50 °C, were prepared. Example 1: DAATFA (10.575 g, 2 mol/L) was dissolved in a small amount of double-distilled water in a pycnometer; then, APS (0.57 g, 10^−1^ mol/L) was added, and the volume was adjusted to 25 mL with double-distilled water (pH 2.5 solution). Aqueous solutions of DAATFA, [M] = 2 mol/L, at several concentrations of the ACVA initiator, [I] = 4 × 10^−2^ and 10^−1^ mol/L, and T = 70 °C, were prepared. Example 2: DAATFA (10.525 g, 2 mol/L) was dissolved in a small amount of double-distilled water in a pycnometer; then, ACVA (0.698 g, 10^−1^ mol/L) was added, and the volume was adjusted to 25 mL with double-distilled water (pH 2.5 solution). The ampoule with the solution was degassed by freezing with liquid nitrogen 10–11 times under vacuum down to 5 × 10^–3^ mm Hg, sealed, and thermostated at 40 or 50 °C. The polymer was isolated in Et_2_O, purified three times by reprecipitation from a solution in MeOH into Et_2_O, and then dried under vacuum over P_2_O_5_. The following samples of PDAATFA were obtained: at 50 °C for t = 30 h, samples P2 and P3; and at 40 °C, sample P4 for 40 h. Samples P5 and P6 were prepared with initiator ACVA at 70 °C for 30 h.

### 3.4. DAATFA RAFT Polymerization

Sample P8 was synthesized in the presence of the RAFT agent xanthate as follows. Radical polymerization of DAATFA was carried out in aqueous solution with initiator ACVA, [M] = 2 mol L^−1^, [ACVA] = 5 × 10^−3^ mol L^−1^, at the ratio of concentrations [xanthate]/[ACVA] = 3, T = 70 °C for 20 h. Sample: DAATFA (10.575 g, 2 mol L^−1^) and xanthate (0.068 g, 1.5 × 10^−2^ mol L^−1^, corresponding to the [xanthate]/[ACVA] = 3) were dissolved in a small amount of bidistilled water; next, initiator ACVA (0.035 g, 5 × 10^−3^ mol L^−1^) and bidistillate were added until the entire volume was 25 mL (pH of solution was 2.5) (see also [21]). The conditions of polymerization and characteristics of the samples are listed in Table 1.

### 3.5. Measurements

^1^H and ^13^C NMR spectra of the synthesized samples were obtained on a Bruker AVANCE III HD spectrometer (400 MHz ^1^H). IR spectra of PDAATFA samples were recorded in ATR reflection mode (ATR) on an IFS-66 v/s Bruker IR spectrometer (ZnSe crystal, scan 30, range 4000–600 cm^−1^). Optical density *D* = lg (*F*_0_/*F*), where *F*_0_ is radiation flux, *F* is flux passed through a layer of matter (Bruker, Billerica, MA, USA).

### 3.6. Determination of Molecular Characteristics of Polymers

The molecular characteristics of the synthesized polymers were determined by hydrodynamic and dynamic light scattering (DLS) methods. The values of the intrinsic viscosity [*η*] of the samples in 1 M NaCl (Ostwald viscometer, solvent flow time 70.5 s) and the translational diffusion coefficients, *D*_0_, were determined according to DLS data (Photokor complex, Moscow, Russia). The viscosity–average molecular weight, *M_η_*, of the samples was calculated using the Mark–Kuhn–Houwink (M-K-H) relation, which we previously obtained for PDAATFA in 1 M NaCl at 298 K [40].

In addition, the experimental values of [*η*] and *D*_0_ of the synthesized samples were used to calculate their hydrodynamic molecular weight, *M_Dη_*, according to Equation (1) [41]:

*M_Dη_* = (A_0_T/*η*_0_*D*_0_)^3^ (100/[*η*]) (1)

where A_0_ is the hydrodynamic invariant, T is the absolute temperature, and *η*_0_ is the viscosity of the solvent. The value of the hydrodynamic invariant A_0_ = 3.0 × 10^−10^ erg/K·mol^1/3^, which is included in Equation (1), for the homologous series of PDAATFA was determined experimentally in [40]. The molecular characteristics of the synthesized samples are given in Table 1. The obtained *M_η_* and *M_Dη_* values correlate well with each other. The methodology of all measurements and formalism are described in detail in [25,40].

### 3.7. Methodology of Toxicity Investigations

Investigations of cytotoxicity were performed using an in vitro method on cell cultures. The dose of the substance in the well was determined, for which a 50% destruction of the cellular monolayer, CTD_50_, was observed. The CTD_50_ is the standard and omnipresent index of toxicity estimation in vitro. It is used as the main parameter in comparative studies of toxicity in all International Programs of toxicity testing in vitro, like ECVAM (EU), CAAT (the USA), and ZEBET (Germany); in databases like TOXLINE or INVITTOX; and in the National Guidelines of Toxicity testing in vitro [42].

In this work, permanent (established) cell lines of eukaryotic cells A-549 (epithelioid line of human lung carcinoma) and MA-104 (epithelioid line of green monkey kidney cells) were used. Cells were grown in the α-MEM cell culture medium (Biolot, St.-Petersburg, Russia) supplemented with 10% calf serum, seeded in 96-well tissue culture plates (Nunc, Roskilde, Denmark), and allowed to grow in the CO_2_ incubator at 5% CO_2_ until the formation of confluent cellular monolayer (usually 24 h). The medium was discarded and replaced with a solution of tested compounds in serial dilutions in the serum-free α-MEM medium. Cells were further incubated for 24 h, and their viability was assessed by the MTT (Thiazolyl blue, Sigma, St. Louis, MO, USA) test [32]. The OD of colored product was measured in ThermoFisher Varioscan Plate Analyzer (Waltham, MA, USA) at 570 nm.

### 3.8. Mathematical/Statistical Analysis of the Results

Each concentration of a compound under study was tested at least in 4 wells of a culture plate (n = 4). Control (intact) cells were represented at n ≥ 4 wells. Each experiment was tripled. CTD_50_ (50% cytotoxic concentration), the concentration which provoked 50% destruction of cellular monolayer, was calculated with the software package GraphPadPrism (GraphPadSoftware, SanDiego, CA, USA, version 6) in the nonlinear regression fit: log(inhibitor) vs. response -- variable slope (four parameters).

### 3.9. Procedure for Antibacterial Activity Research

Standard reference strains were used for polymer activity testing, namely *Pseudomonas aeruginosa* (ATCC 27853) and *Staphylococcus aureus* (ATCC 25923) obtained from the State Collection of Pathogenic Microorganisms and Cell Cultures of State Research Center for Applied Microbiology and Biotechnology, Russia. Bacteria *P*. *aeruginosa* and *S*. *aureus* were grown in NB medium (Himedia, Thane, India) for 20 h. Bacterial inoculums were adjusted with sterile NB medium to a 1 McFarland standard with an organism density of approximately 3 × 10^8^ colony-forming units (CFU)/mL. Then, the suspension was diluted with NB broth to make a 1:3000 bacterial dilution (1 × 10^5^ CFU/mL).

### 3.10. Estimation of Bacterial Viability

Bacteria *P*. *aeruginosa* and *S*. *aureus* were then inoculated at a concentration of 10^5^ CFU/mL into 15 mL test tubes containing 2 mL of NB medium (Himedia, Thane, India) and polymer aqueous solutions of different concentrations prepared by serial dilutions (500 μg·mL^−1^ was the maximal initial concentration of the polymer solutions to be investigated). After 24 h of incubation at 37 °C and 120 r.p.m., the culture from each tube was spread on agar-solidified NB medium by the streak-seeding method and incubated at 37 °C. The viability of bacteria was determined after 2 days (presence or absence of bacterial growth all along the streak), and the minimal bactericidal concentrations corresponding to each treatment time (MBC_100_, or MBC) were determined, i.e., concentration required to eliminate detectable growth of cells. The detection limit of the spread-plate method, using a 100 µL plating volume, was estimated between 10 and 30 CFU·mL^−1^ compared to the initial 10^5^ CFU·mL^−1^. All experiments were carried out at least 4 times, and the data are reported as the mean values ± ER (experimental errors, which were calculated according to the recommended procedures).

## 4. Conclusions

Summarizing the obtained results on the effect of the end groups of PDAATFA polymers with a medium and low MW on their bactericidal activity and cytotoxicity relative to eukaryotic cells, we can conclude that the dependence on MW of the activity and, most of all, toxicity is preserved even at a small difference in MW values in the MW range of (18–40) × 10^3^ g·mol^−1^. Secondly, a clear dependence of the studied properties on the nature of the end groups was revealed. Sulfate -O-S(=O)2-O¯ end group has a noticeable effect on the bactericidal efficiency and smaller influence on toxicity, while dithiocarbonyl end group -S-C(=S)-O-CH_2_-CH_3_ has a significant effect on efficiency and especially toxicity, drastically increasing the latter.

Due to the protonated ammonium groups in pyrrolidinium links, polymers PDAAs have advantages over quaternary low-molecular-weight and polymer compounds [2,3,35,36,37]. First of all, this is manifested in the low MBC values that can allow us to use the effective low-toxic solutions of low concentrations (especially relative to *St. aureus*). The protonated form of PDAAs allows us to use solutions of small and medium ionic strength (serum, 0.01M/0.1M, as mentioned above), and aqueous alkaline solutions, as is important for practical bio-medical applications.

The results of investigations of the bioactive properties of low-MW PDAA (especially 18 × 10^3^ g·mol^−1^), for which selectivity is about 1.16 (relative to *St. aureus*), seem to be hopeful. To reduce its (and polymers with lower MW) cytotoxic effect, a low inhibitory concentration should be used in order to achieve the effect of total death of bacterial cells in a longer period of time (two to three days). Another way to reduce cytotoxicity is the creation of polycomplexes formed by polyanion–polycation pairs, for example, anionic sodium alginate–PDAA. Polycomplexes of natural compounds are of especial interest due to their low toxicity, high biocompatibility, and biodegradability [43,44].

The data obtained allow us to plan in vivo studies to evaluate the antimicrobial effect of polymers on the diseased organism. Overall, based on the results obtained, we consider PDAA polymers of low MW to be promising antimicrobial agents for the creation of new transdermal drugs.

## Data Availability

The original contributions presented in this study are included in the article. Further inquiries can be directed to the corresponding authors.

## References

[1] Kyzioł A., Khan W., Sebastian V., Kyzioł K. (2020). Tackling microbial infections and increasing resistance involving formulations based on antimicrobial polymers. Chem. Eng. J..

[2] Siedenbiedel F., Tiller J.S. (2012). Antimicrobial polymers in solution and on surfaces: Overview and functional principles. Polymers.

[3] Chen A., Peng H., Blakey I., Whittaker A.K. (2017). Biocidal Polymers: A Mechanistic Overview. Polym. Rev..

[4] Palermo E.F., Lienkamp K., Gilles E.R., Ragogna P.J. (2019). Antibacterial activity of polymers: Discussions on the nature of amphiphilic balance. Angew. Chem. Int. Ed..

[5] Ergene C., Yasuhara K., Palermo E.F. (2018). Biomimetic antimicrobial polymers: Recent advances in molecular design. Polym. Chem..

[6] Sarapas J.M., Backlund C.M., deRonde B.M., Minter L.M., Tew G.N. (2017). ROMP- and RAFT-Based Guanidinium-Containing Polymers as Scaffolds for Protein Mimic Synthesis. Chem.-Eur. J..

[7] Boschert D., Schneider-Chaabane A., Himmelsbach A., Eickenscheidt A., Lienkamp K. (2018). Synthesis and Bioactivity of Polymer-Based Synthetic Mimics of Antimicrobial Peptides (SMAMPs) Made from Asymmetrically Disubstituted Itaconates. Chem.-Eur. J..

[8] Takahashi H., Palermo E.F., Yasuhara K., Caputo G.A., Kuroda K. (2013). Molecular Design, Structures, and Activity of Antimicrobial Peptide-Mimetic Polymers. Macromol. Biosci..

[9] Oda Y., Yasuhara K., Kanaoka S., Sato T., Aoshima S., Kuroda K. (2018). Aggregation of Cationic Amphiphilic Block and Random Copoly(vinyl ether)s with Antimicrobial Activity. Polymers.

[10] Krumm C., Trump S., Benski L., Wilken J., Oberhaus F., Kцller M., Tiller J.C. (2020). Fast-Acting Antibacterial, Self-Deactivating Polyionene Esters. ACS Appl. Mater. Interfaces.

[11] Timofeeva L.M., Vasilieva Y.A., Klescheva N.A., Gromova G.L., Timofeeva G.I., Rebrov A.I., Topchiev D.A. (2002). Synthesis of high-molecular-weight polymers based on N, N-diallyl-N-methylamine. Macromol. Chem. Phys..

[12] Timofeeva L.M., Klescheva N.A., Vasilieva Y.A., Gromova G.L., Timofeeva G.I., Filatova M.P. (2005). Mechanism and kinetic features of producing new polymers based on monomers of diallylamine series. Polym. Sci. Ser. A.

[13] Timofeeva L.M., Klescheva N.A., Moroz A.F., Didenko L.V. (2009). Secondary and tertiary polydiallylammonium salts: Novel polymers with high antimicrobial activity. Biomacromolecules.

[14] Timofeeva L.M., Klescheva N.A., Shleeva M.O., Filatova M.P., Simonova Y.A., Ermakov Y.A., Kaprelyants A.S. (2015). Nonquaternary poly(diallylammonium) polymers with different amine structure and their biocidal effect on *Mycobacterium tuberculosis* and *Mycobacterium smegmatis*. Appl. Microbiol. Biotechnol..

[15] Rutala W.A., Weber D.J., Bennett J.E., Dolin R., Blaser M.J. (2020). Disinfection, Sterilization, and Control of Hospital Waste. Book Mandell, Douglas, and Bennett’s Principles and Practice of Infectious Diseases.

[16] Timofeeva L., Bondarenko G., Nikitushkin V., Simonova Y., Topchiy M., Eremenko I., Shleeva M., Mulyukin A., Kaprelyants A. (2022). On the molecular mechanism of nonspecific antimicrobial action of protonated diallylammonium polymers on mycobacterial cells. Europ. Polym. J..

[17] Li Petri G., Raimondi M.V., Spano V., Holl R., Barraja P., Montalbano A. (2021). Pyrrolidine in Drug Discovery: A Versatile Scaffold for Novel Biologically Active Compounds. Top. Curr. Chem..

[18] Raimondi M.V., Listro R., Cusimano M.G., La Franca M., Faddetta T., Gallo G., Schillaci D., Collina S., Leonchiks A., Barone G. (2019). Pyrrolomycins as antimicrobial agents. Microwave-assisted organic synthesis and insights into their antimicrobial mechanism of action. Bioorg. Med. Chem..

[19] Simonova Y.A., Eremenko I.V., Topchiy M.A., Kozobkova N.V., Shleeva M.O., Eropkin M.Y., Timofeeva L.M. (2025). Antimicrobial protonated polydiallylamines: How to retain bactericidal efficiency at minimal toxicity. Mendeleev Commun..

[20] Chiefari J., Chong Y.K., Ercole F., Krstina J., Jeffery J., Le T.P.T., Mayadunne R.T.A., Meijs G.F., Moad C.L., Moad G. (1998). Living-Free radical polymerization by reversible addition–fragmentation chain transfer: The RAFT process. Macromolecules.

[21] Destarac M., Bzducha W., Taton D., Gauthier-Gillaizeau I., Zard S.Z. (2002). Xanthates as chain-transfer agents in controlled radical polymerization (MADIX): Structural effect of the O-alkyl group. Macromol. Rapid Commun..

[22] Barner-Kowollik C. (2008). Handbook of RAFT Polymerization.

[23] Moad G., Chong Y.K., Postma A., Rizzardo E., Thang S.H. (2005). Advances in RAFT polymerization: The synthesis of polymers with defined end-groups. Polymer.

[24] Michl T.D., Locock K.E.S., Stevens N.E., Hayball J.D., Vasilev K., Postma A., Qu Y., Traven A., Haeussler M., Meagher L. (2014). RAFT-derived antimicrobial polymethacrylates: Elucidating the impact of end-groups on activity and cytotoxicity. Polym. Chem..

[25] Simonova Y.A., Topchiy M.A., Filatova M.P., Yevlampieva N.P., Slyusarenko M.A., Bondarenko G.N., Asachenko A.F., Nechaev M.S., Timofeeva L.M. (2020). Impact of the RAFT/MADIX agent on protonated diallylammonium monomer cyclopolymerization with efficient chain transfer to monomer. Eur. Polym. J..

[26] Timofeeva L.M., Vasilieva Y.A., Klescheva N.A., Gromova G.L., Topchiev D.A. (2002). Radical polymerization of diallylamine compounds: From quantum chemical modeling to controllable synthesis of high-molecular-weight polymers. Int. J. Quantum. Chem..

[27] Eremenko I.V., Simonova Y.A., Filatova M.P., Yevlampieva N.P., Bondarenko G.N., Kleshcheva N.A., Timofeeva L.M. (2024). Optimization of methodology of protonated diallylammonium monomers free radical polymerization for the obtaining polymers with a low molecular weight. Russ. J. Appl. Chem..

[28] Simonova Y.A., Filatova M.P., Timofeeva L.M. (2018). Radical Polymerization of Protonated Diallylammonium Monomers in Bidistilled Aqueous Solution: Kinetic Study. Polym. Sci. Ser. B.

[29] Seibert H., Balls M., Fentem J.H., Bianchi V., Clothier R.H., Dierickx P.J., Ekwall B., Garle M.J., Gómez-Lechón M.J., Gribaldo L. (1996). Acute Toxicity Testing in Vitro and the Classification and Labelling of Chemicals: The Report and Recommendations of ECVAM Workshop 16. Altern. Lab. Anim..

[30] Clemedson C., Ekwall B. (1999). Overview of the final MEIC results. I. The in vitro- in vitro evaluation. Toxicol. In Vitro.

[31] Balls M., Worth A.P., Balls M., Combes R., Worth A.P. (2019). The History of Alternative Test Methods in Toxicology.

[32] Borenfreund E., Babich H., Martin-Alguicil N. (1988). Comparison of two in vitro cytotoxicity assays—The neutral red (NR) and tetrazolium (MTT) tests. Toxicol. In Vitro.

[33] Kwon S., Yang W., Moon D., Kim K.S. (2020). Comparison of Cancer Cell Elasticity by Cell Type. J. Cancer.

[34] Ikeda T., Hirayama H., Yamaguchi H., Tazuke S., Watanabe M. (1986). Polycationic Biocides with Pendant Active Groups: Molecular Weight Dependence of Antibacterial Activity. Antimicrob. Agents. Chemother..

[35] Kenawy E.-R., Worley S.D., Broughton R. (2007). The Chemistry and Applications of Antimicrobial Polymers: A State-of-the-Art Review. Biomacromolecules.

[36] Timofeeva L., Kleshcheva N. (2011). Antimicrobial polymers: Mechanism of action, factors of activity and applications. Appl. Microbiol. Biotechnol..

[37] Jain A., Duvvuri L.S., Farah S., Beyth N., Domb A.J., Khan W. (2014). Antimicrobial polymers. Adv. Healthc. Mater..

[38] McDonnell G., Russell A.D. (1999). Antiseptics and disinfectants: Activity, action, and resistance. Clin. Microbiol. Rev..

[39] Franklin T.J., Snow G.A. (2005). Biochemistry and Molecular Biology of Antimicrobial Drug Action.

[40] Yevlampieva N., Vezo O., Simonova Y., Timofeeva L. (2018). Protonated Member of Poly(diallylammonium) Family: Hydrodynamic and Conformational Properties. Int. J. Polym. Anal. Character.

[41] Tsvetkov V.N. (1989). Rigid-Chain Polymers.

[42] GOST MU 1.2.2635-10; Rospotrebnadzor: 2010; 122p. https://www.russiangost.com/p-146565-mu-122635-10.aspx.

[43] Fay J.M., Kabanov A.V. (2022). Interpolyelectrolyte Complexes as an Emerging Technology for Pharmaceutical Delivery of Polypeptides. Rev. Adv. Chem..

[44] Sinelnikova D.G., Novoskoltseva O.A., Loiko N.G., Nikolaev Y.A., Yaroslavov A.A. (2024). Hybrid polycomplexes of anionic alginate with a synthetic cationic polymer: Attractive and poisonous for microorganisms. Mendeleev Commun..

